# Corrigendum: Beta 1-integrin–c-Met cooperation reveals an inside-in survival signalling on autophagy-related endomembranes

**DOI:** 10.1038/ncomms12392

**Published:** 2016-07-22

**Authors:** Rachel Barrow-McGee, Naoki Kishi, Carine Joffre, Ludovic Ménard, Alexia Hervieu, Bakhouche A. Bakhouche, Alejandro J. Noval, Anja Mai, Camilo Guzmán, Luisa Robbez-Masson, Xavier Iturrioz, James Hulit, Caroline H. Brennan, Ian R. Hart, Peter J. Parker, Johanna Ivaska, Stéphanie Kermorgant

Nature Communications 7 Article number:1194210.1038/ncomms11942 (2016); Published 07 23 2016; Updated 07 21 2016

The original version of this Article contained an error in the spelling of the author Luisa Robbez-Masson, which was incorrectly given as Luisa Robert-Masson. This has now been corrected in both the PDF and HTML versions of the Article.

