# Supplementary material for: Beta 1-integrin–c-Met cooperation reveals an inside-in survival signalling on autophagy-related endomembranes
Source: Nat Commun. 2016 Jun 23;7:11942. doi: 10.1038/ncomms11942 (PMC4931016; doi:10.1038/ncomms11942)
Supplement: Supplementary Information — Supplementary Figures 1-11 and Supplementary Table 1 [file ncomms11942-s1.pdf]

Supplementary Information

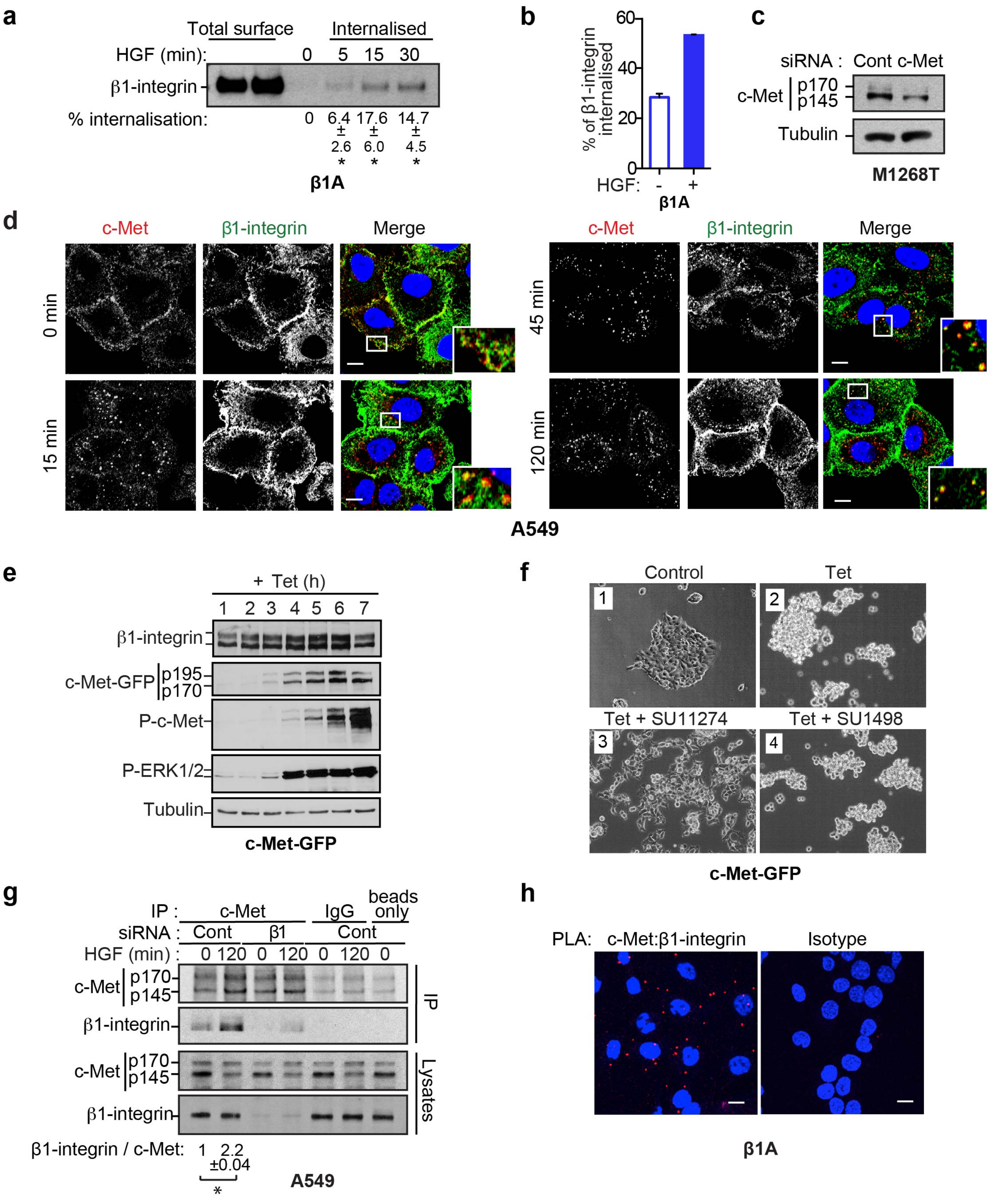

Supplementary Figure 1 (related to Figure 1): c-Met and β1-integrin co-internalise in a molecular complex in cells both adherent and in suspension

a) Western blot for β1-integrin following a biotinylation internalisation assay in β1A cells, which were incubated with HGF for 5, 15 and 30 min at 37°C. Numbers are mean percentages of internalisation ± SEM (n=3).

b) The mean percentage of β1-integrin internalisation, obtained by biotinylation internalisation assay, in β1A cells incubated at 37°C for 15 min without (-) or with (+) HGF compared to total cell surface β1-integrin levels ± range of error (n=2).
c) Western blots for c-Met and tubulin in M1268T c-Met expressing NIH3T3 cells transfected with control or c-Met siRNA.
d) Confocal sections of A549 cells stimulated with HGF for 0, 15, 45 and 120 min and stained for c-Met (red), β1-integrin (green) and DAPI (blue). Colocalisations appear in yellow. Bars: 10 μm.
e) Western blots for β1-integrin, GFP (c-Met-GFP: p195, precursor; p170, mature β chain), phospho-c-Met (Y1234-35), phospho-ERK1/2 and tubulin in c-Met-GFP cells, incubated with tetracycline (Tet) for the times indicated.
f) Low light images of c-Met-GFP cells, incubated or not (Control) with tetracycline (Tet) for 16 h and further incubated for 1 h or not with the c-Met inhibitor SU11274 (2 μM) or the unrelated VEGFR2 inhibitor SU1498 (10 μM).
g) Western blots for c-Met and β1-integrin following immunoprecipitation with c-Met CVD13 antibody, IgG control or no antibody (beads only) in A549 cells. Cells, transfected with control (Cont) or β1-integrin (β1) siRNA, were stimulated with HGF for 0 or 120 min. Total c-Met and β1-integrin levels in the cell lysates are shown. Numbers ± SEM (n=3) represent the levels of β1-integrin co-immunoprecipitated, normalised to c-Met immunoprecipitate, at 0 min (levels set as 1) and 120 min of HGF stimulation (levels expressed as a fold change from 0 min). Values, obtained by densitometric analysis, were first tresholded on IgG values.
h) Proximity Ligation Assay. Confocal sections of β1A cells stained with c-Met and β1-integrin or IgG isotypes, followed by the binding of PLA probes. Red dots indicate β1-integrin-c-Met proximity. Bars: 10 μm.
* $p<0.05$

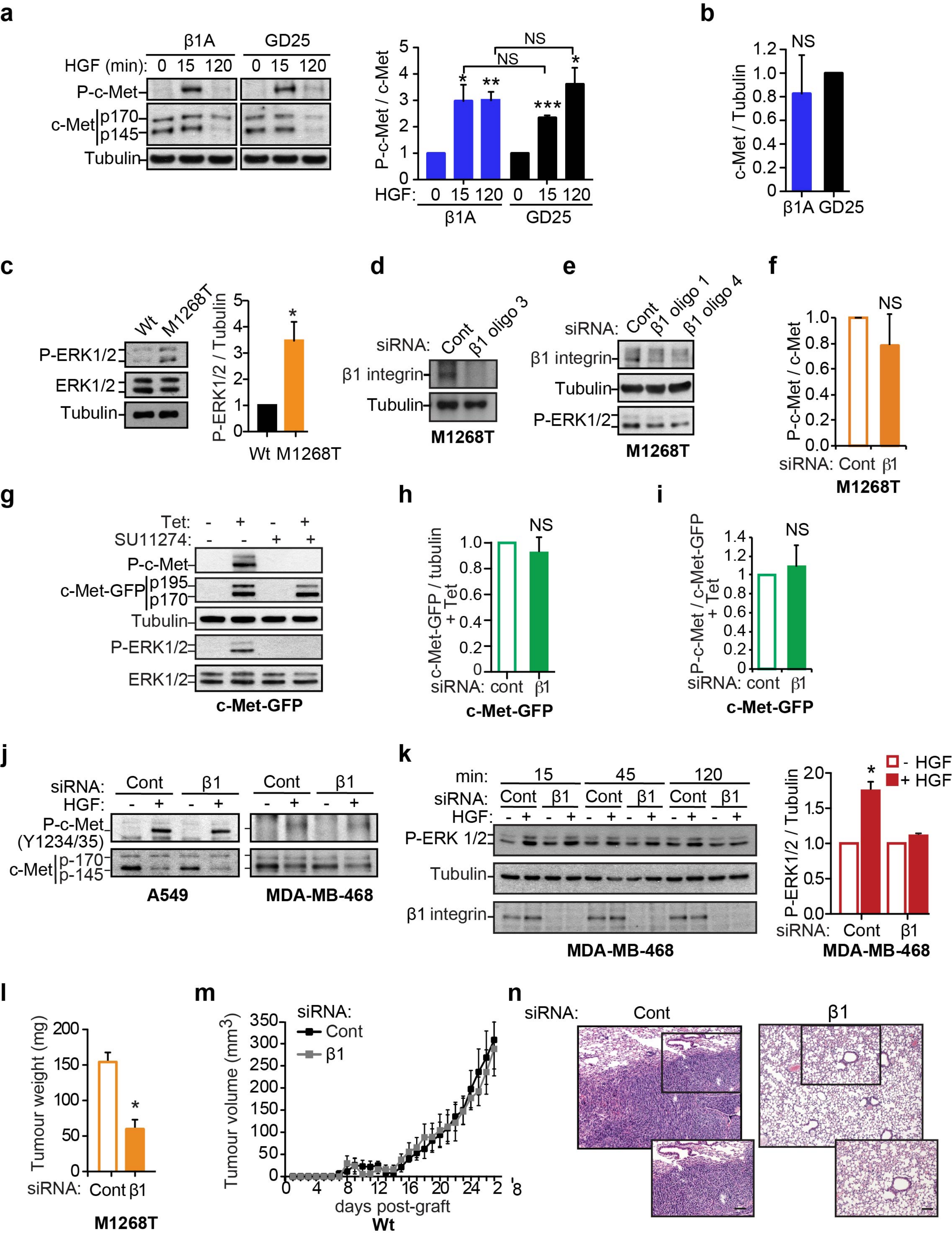

a
β1A
GD25
HGF (min): 0 15 120 0 15 120
P-c-Met
c-Met
p170
p145
Tubulin
P-c-Met / c-Met
NS
NS
*
**
***
*
HGF: 0 15 120 0 15 120
β1A
GD25
b
NS
c-Met / Tubulin
β1A GD25
c
Wt
M1268T
P-ERK1/2
ERK1/2
Tubulin
P-ERK1/2 / Tubulin
*
Wt M1268T
d
siRNA: Cont β1 oligo 3
β1 integrin
Tubulin
M1268T
e
siRNA: Cont β1 oligo 1 β1 oligo 4
β1 integrin
Tubulin
P-ERK1/2
M1268T
f
NS
P-c-Met / c-Met
siRNA: Cont β1
M1268T
g
Tet: - + - +
SU11274: - - + +
P-c-Met
c-Met-GFP
p195
p170
Tubulin
P-ERK1/2
ERK1/2
c-Met-GFP
h
NS
c-Met-GFP / tubulin + Tet
siRNA: cont β1
c-Met-GFP
i
NS
P-c-Met / c-Met-GFP + Tet
siRNA: cont β1
c-Met-GFP
j
siRNA: Cont β1 Cont β1
HGF: - + - + - + - +
P-c-Met (Y1234/35)
c-Met
p-170
p-145
A549
MDA-MB-468
k
min: 15 45 120
siRNA: Cont β1 Cont β1 Cont β1
HGF: - + - + - + - + - + - +
P-ERK 1/2
Tubulin
β1 integrin
MDA-MB-468
- HGF
+ HGF
*
P-ERK1/2 / Tubulin
siRNA: Cont β1
MDA-MB-468
l
Tumour weight (mg)
*
siRNA: Cont β1
M1268T
m
siRNA:
Cont
β1
Tumour volume (mm3)
days post-graft
Wt
n
siRNA: Cont β1

o

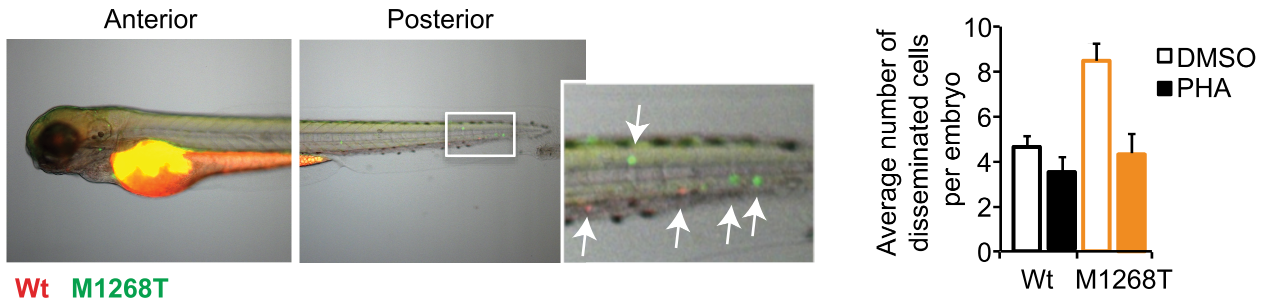

Supplementary Figure 2 (related to figure 2): β1-integrin is required for sustained c-Met-dependent ERK1/2 phosphorylation in detached cells, c-Met-dependent in vivo tumourigenesis, invasion and lung metastasis.

a) Western blots for phospho-c-Met (Y1234-35), c-Met and tubulin in β1A cells stimulated with HGF for 0, 15 or 120 min. Graph represents the mean phospho-c-Met / c-Met ratios ± SEM with HGF normalised to the ratios obtained without HGF, obtained by densitometry (n=4).

b) Mean c-Met / tubulin ratios (n=3) ± SEM in β1A cells normalised to the ratios obtained in GD25 cells, following densitometry analyses of Western blots (shown in Fig. S2a).

c) Western blots for phospho-ERK1/2, ERK1/2 and tubulin in Wt and M1268T c-Met expressing NIH3T3 cells. Graph represents the mean phospho-ERK1/2 / tubulin ratios ± SEM in M1268T c-Met expressing NIH3T3 cells normalised on the ratios in Wt cells, obtained by densitometry (n=3).d) Western blot for β1-integrin and tubulin in M1268T c-Met expressing NIH3T3 cells transfected with Control (Cont) or β1-integrin (β1) siRNA (oligo 3, used in Figure 2b).

e) Western blot for β1-integrin, tubulin and phospho-ERK1/2 in M1268T c-Met expressing NIH3T3 cells transfected with Control (Cont) or two individual β1-integrin (β1) siRNA oligos (1 and 4).

f) Graph represents the mean phospho-c-Met / c-Met ratios ± SEM in M1268T c-Met expressing NIH3T3 cells transfected with β1-integrin (β1) siRNA normalised to the ratios in cells transfected with Control (Cont) siRNA. Data obtained by densitometry of Western blots (shown Fig. 2b, n=3).

g) Western blots for phospho-c-Met (Y1234-35), GFP (c-Met-GFP: p195, precursor; p170, mature β chain), phospho-ERK1/2, ERK1/2 and tubulin in c-Met-GFP cells, incubated without (-) or with (+) tetracycline (Tet) for 16 h and further treated (+) or not (-) with the c-Met inhibitor SU11274 (2 μM) for 1 h.

hi) Graphs represent the mean ratios ± SEM of (h) p170 c-Met-GFP / tubulin and (i) phospho-c-Met / c-Met-GFP in c-Met-GFP cells transfected with β1-integrin (β1) siRNA and incubated with tetracycline (Tet) for 16 h, normalised to corresponding ratios obtained in cells transfected with control (Cont) siRNA. Data obtained by densitometry of Western blots (shown Fig. 2c, n=3)

j) Western blots for phospho-c-Met (Y1234-35) and c-Met in A549 cells (left panel) and MDA-MB-468 cells (right panel), transfected with control (Cont) or β1-integrin (β1) siRNA siRNA and stimulated without (-) or with (+) HGF for 120 min in suspension.

k) Western blots for phospho-ERK1/2, tubulin and β1-integrin in MDA-MB-468 cells, transfected with control (Cont) or β1-integrin (β1) siRNA and stimulated without (-) or with (+) HGF for 15, 45 or 120 minutes in suspension. Graph represents mean phospho-ERK1/2 / tubulin ratios ± SEM with HGF normalised to no HGF, obtained by densitometric analysis (n=3).

l) Mean weight (mg) of tumours derived from M1268T c-Met expressing NIH3T3 cells transfected with control (Cont) or β1-integrin (β1) siRNA, at day 11 ± SEM (n=5 mice per group).

m) Tumour growth curve, over time, of Wt c-Met expressing NIH3T3 cells, transfected with control (Cont) or β1-integrin (β1) siRNA. Data are mean tumour volume (mm3) ± SEM (n=5 mice in control siRNA group, n=4 mice in β1-integrin siRNA group).

n) Representative histological Haematoxylin & Eosin stained sections of the lungs of mice injected in the tail vein with M1268T c-Met expressing NIH3T3 cells transfected with control (Cont) or β1-integrin (β1) siRNA. Bar: 200 μm.

o) Left panel: pictures of the anterior and posterior section of 72 h old zebrafish embryos that had been injected 24 h previously with Wt (green) and M1268T (red) c-Met expressing NIH3T3 cells. Disseminated cells in the body of the embryo are labeled with white arrows. Right panel: average number of disseminated Wt and M1268T c-Met expressing NIH3T3 cells, treated with DMSO or PHA-665752 (PHA, 100 nM), per embryo 24 h after injection ($n=1$, average of 29 embryos per condition).

* $p<0.05$; **$p<0.01$; ***$p<0.001$; NS: not significant.

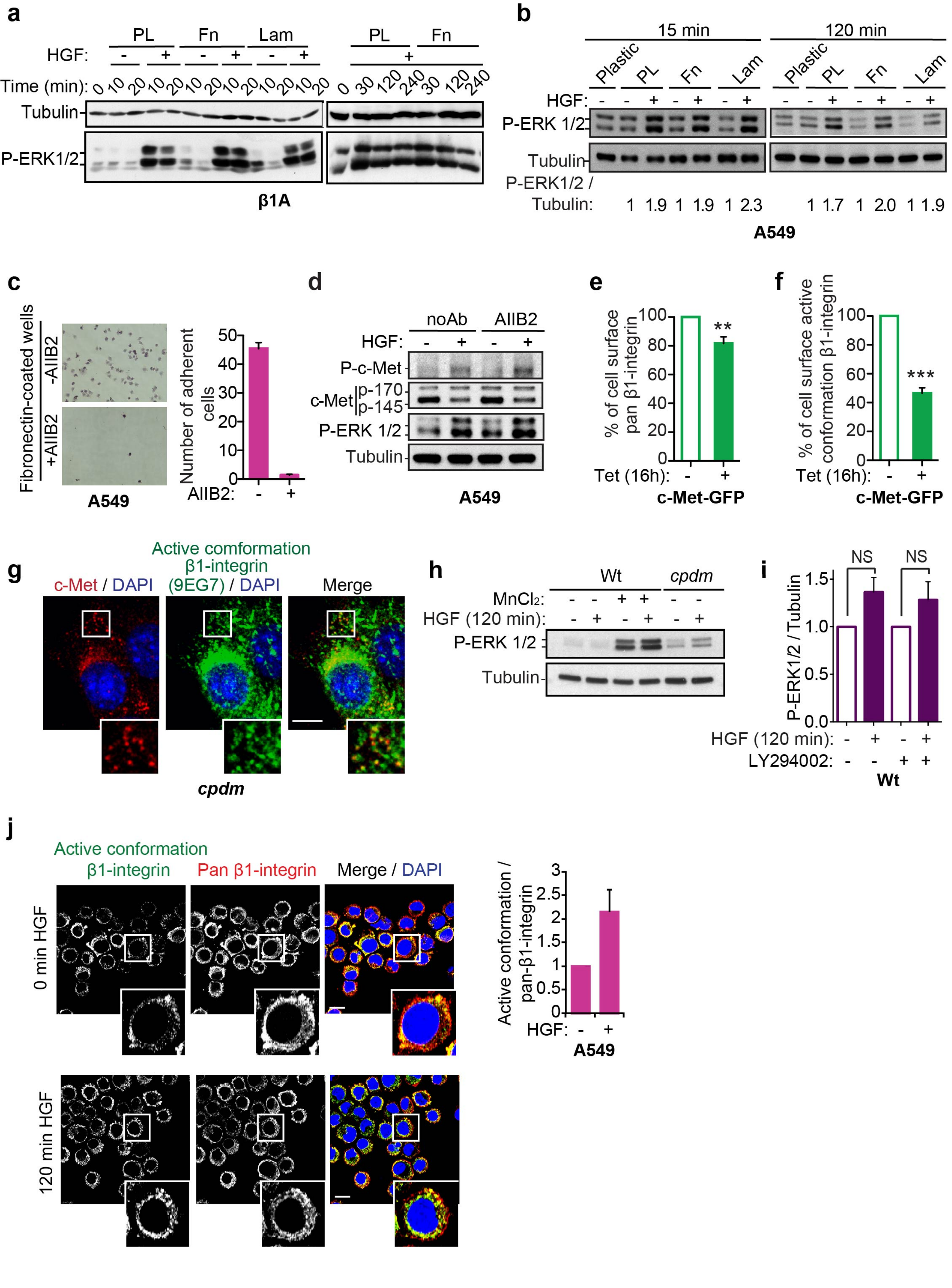

a
PL Fn Lam PL Fn
HGF: - + - + - + +
Time (min): 0 10 20 10 20 10 20 10 20 10 20 10 20 0 30 120 240 30 120 240
Tubulin
P-ERK1/2
β1A
b
15 min 120 min
Plastic PL Fn Lam Plastic PL Fn Lam
HGF: - - + - + - + - - + - + - +
P-ERK 1/2
Tubulin
P-ERK1/2 / Tubulin: 1 1.9 1 1.9 1 2.3 1 1.7 1 2.0 1 1.9
A549
c
Fibronectin-coated wells
-AIIB2
+AIIB2
A549
Number of adherent cells
AIIB2: - +
d
noAb AIIB2
HGF: - + - +
P-c-Met
c-Met p-170 p-145
P-ERK 1/2
Tubulin
A549
e
% of cell surface pan β1-integrin
Tet (16h): - +
c-Met-GFP
f
% of cell surface active conformation β1-integrin
Tet (16h): - +
c-Met-GFP
g
c-Met / DAPI
Active comformation β1-integrin (9EG7) / DAPI
Merge
cpdm
h
Wt cpdm
MnCl2: - - + + - -
HGF (120 min): - + - + - +
P-ERK 1/2
Tubulin
i
NS NS
P-ERK1/2 / Tubulin
HGF (120 min): - + - +
LY294002: - - + +
Wt
j
Active conformation β1-integrin
Pan β1-integrin
Merge / DAPI
0 min HGF
120 min HGF
Active conformation / pan-β1-integrin
HGF: - +
A549

Supplementary Figure 3 (related to Figure 3): The role of β1-integrin in c-Met-dependent signalling is adhesion-independent though its active conformation is a positive regulator
a) Western blots for phospho-ERK1/2 and tubulin in β1A cells non plated (0) or plated on poly-L-lysine (PL), fibronectin (Fn) or laminin (Lam) for the indicated times with (+) or without (-) HGF.
b) Western blots for phospho-ERK 1/2 and tubulin in A549 cells, plated on plastic, poly-l-lysine (PL), fibronectin (FN) or laminin (Lam) and stimulated without (-) or with (+) HGF for 15 or 120 min. Numbers represent phospho-ERK1/2 / tubulin ratios upon HGF normalised to the ratios with no HGF, obtained by densitometry (n=1).
c) Left panel: images of adherent A549 cells to fibronectin following treatment with or without AIIB2 β1-integrin blocking antibody. Right panel: average number of adherent A549 cells on fibronectin following treatment with (+) or without (-) AIIB2 β1-integrin blocking antibody (n=1, performed in triplicate).
d) Western blots for phospho-c-Met (Y1234-35), c-Met, phospho-ERK1/2 and tubulin in A549 cells treated with or without AIIB2 β1-integrin blocking antibody for 15 min prior to stimulation without (-) or with (+) HGF for 120 min in suspension.
ef) Mean percentage of cell surface levels ± SEM of (e) pan-β1-integrin (DF7) or (f) active-conformation β1-integrin (9EG7), obtained by flow cytometry, in GFP-c-Met cells in the presence of tetracycline (Tet) for 16h normalised to the % without tetracycline (Tet) (n=4).
g) Confocal section of cpdm (Sharpin null) cells stimulated with HGF for 120 min, fixed and stained for c-Met (red), active conformation β1-integrin (9EG7, green) and DAPI (blue). Bar: 10 μm.
h) Western blots for phospho-ERK1/2 and tubulin in Wt Sharpin MEFs treated with and without MnCl2 (1 mM) and cpdm (Sharpin null) cells stimulated without (-) and with (+) HGF for 120 min in suspension.
i) Mean phospho-ERK1/2 / tubulin ratio ± SEM in Wt MEFs, stimulated without (-) or with (+) HGF for 120 min in suspension and treated without (-) or with (+) LY294002 (10 μM) (n=3).
j) Confocal sections of A549 cells stimulated with or without HGF for 120 min in suspension. Cells were cytospun and stained for active-conformation β1-integrin (green), pan-β1-integrin (red) and DAPI (blue). Co-localisation between active and pan-β1-integrin appears in yellow. The graph represents the mean proportion ± range of error of active-conformation β1-integrin pixels over pan-β1-integrin pixels with HGF, normalised to no HGF (n=2). Bars: 10 μm.
**p<0.01; ***p<0.001.

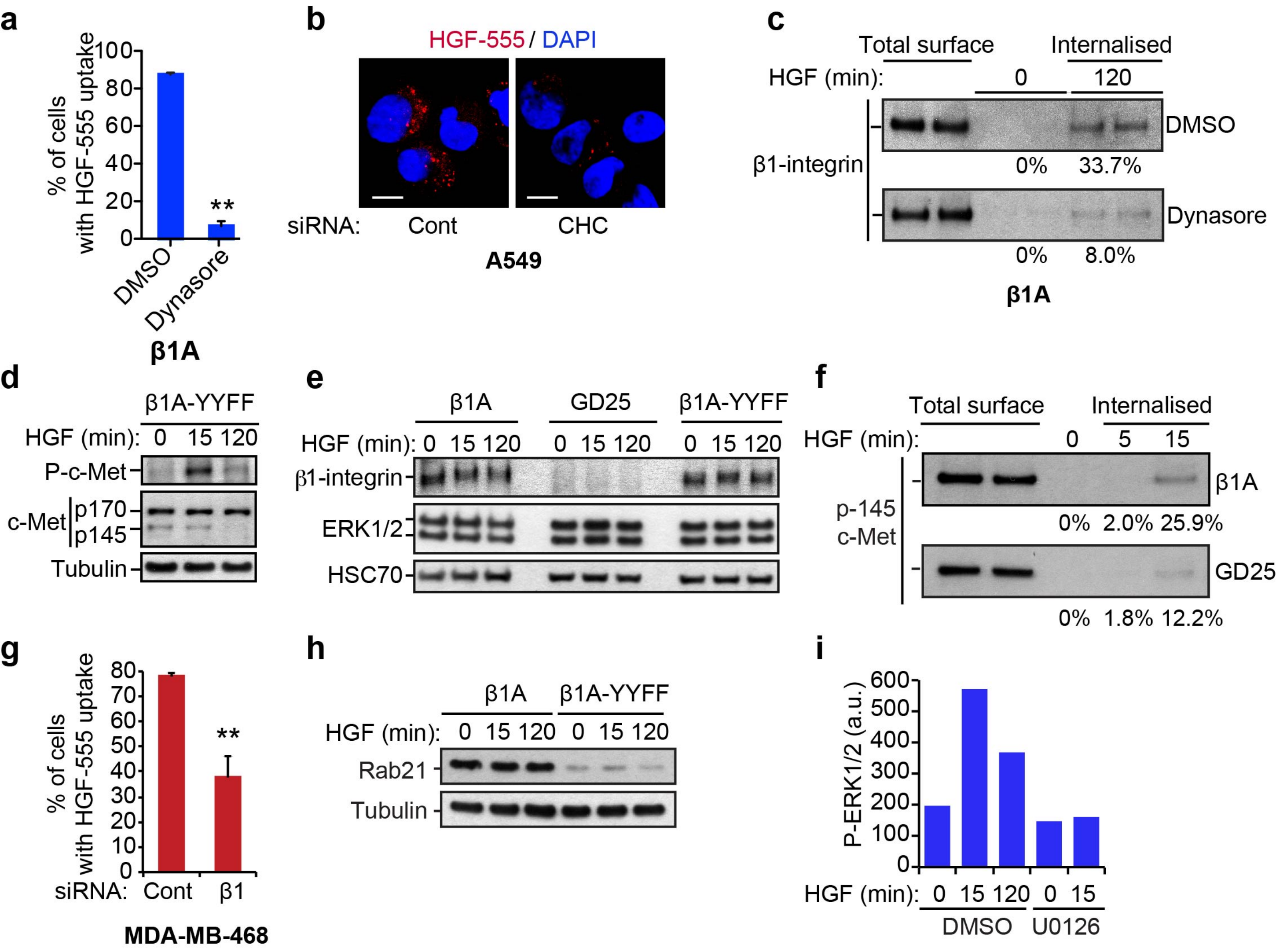

Supplementary Figure 4 (related to Figure 4): c-Met and β1-integrin cooperation is endocytosis-dependent and β1-integrin is required for c-Met endocytosis

a) Mean percentage ± SEM of β1A cells that contained HGF-AlexaFluor-555 (HGF-555) after 120 min stimulation following pre-treatment with DMSO or Dynasore (80 μM). (n=3).

b) Confocal sections of A549 cells stimulated with HGF-AlexaFluor-555 (HGF-555, red) for 120 min in suspension and stained for DAPI (blue) following transfection with control (Cont) or clathrin heavy chain (CHC) siRNA. Bars: 10 μm.

c) Western blot for β1-integrin following a biotinylation internalisation assay in β1A cells incubated at 37°C for 0 or 120 min following pre-treatment with DMSO or Dynasore (80 μM). Numbers are the percentage of internalisation (n=1).

d) Western blots for phospho-c-Met (Y1234-35), c-Met and tubulin in β1A-YYFF cells, stimulated with HGF for 0, 15 and 120 min.

e) Western blots for β1-integrin, ERK1/2 and HSC70 in β1A, GD25 and β1A-YYFF cells stimulated with HGF for 0, 15 and 120 min.

f) Western blots for c-Met following a biotinylation internalisation assay in GD25 and β1A cells. Cells were incubated at 37°C for 0, 5, and 15 min. Numbers are the percentage of internalisation (n=1).

g) Mean percentage ± SEM of MDA-MB-468 cells that contained HGF-AlexaFluor-555 (HGF-555) after stimulation for 120 min following transfection with control (Cont) or β1-integrin (β1) siRNA (n=3).

h) Western blot for Rab21 and tubulin in β1A and β1A-YYFF cells stimulated with HGF for 0, 15 and 120 min.

i) Level of phospho-ERK1/2 detected by flow cytometry in β1A cells upon HGF stimulation (fluorescence intensity, in arbitrary units, n=1). Cells were treated with DMSO or the MEK inhibitor U0126 (10 μm).

**p<0.01.

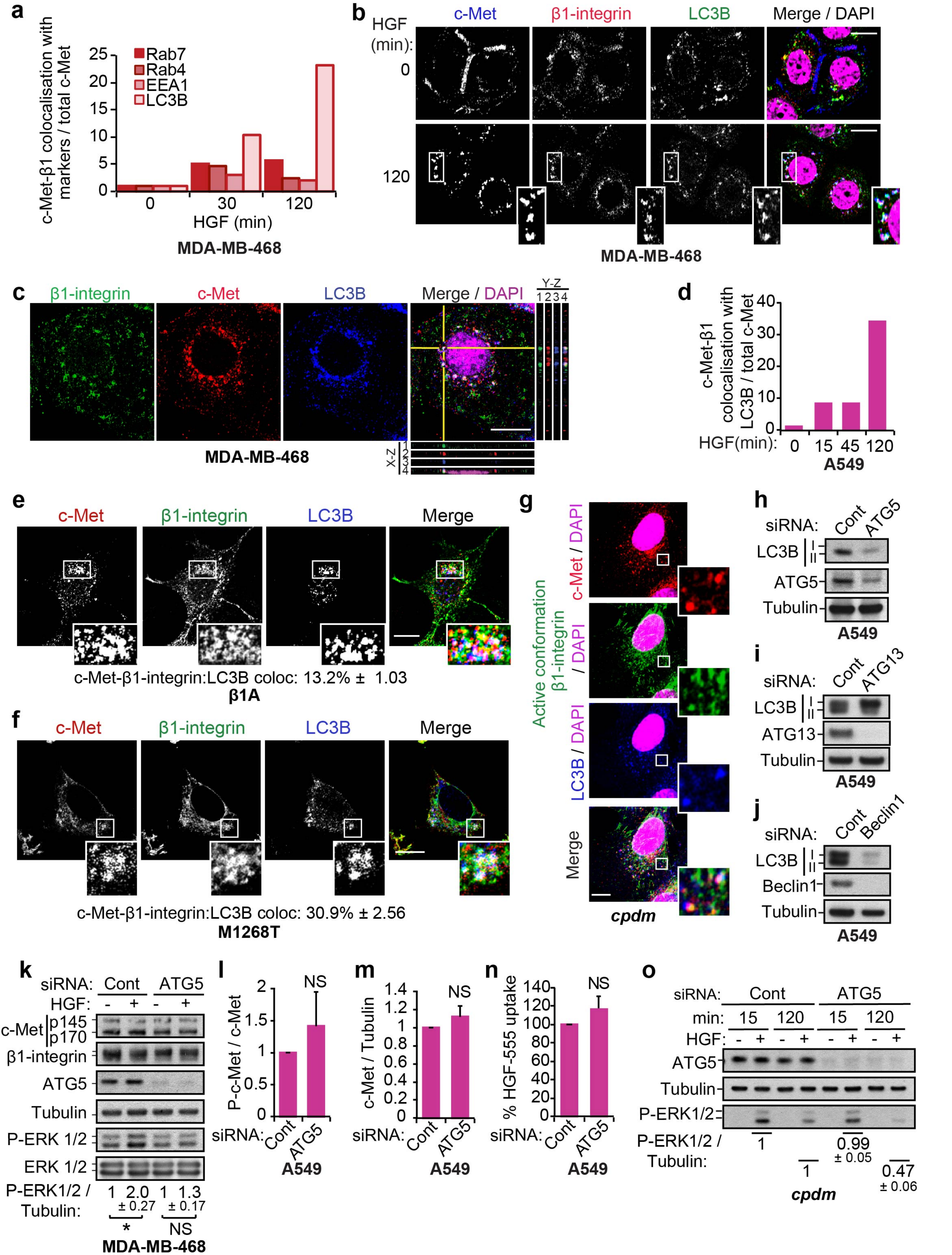
a
c-Met-β1 colocalisation with markers / total c-Met
Rab7
Rab4
EEA1
LC3B
HGF (min)
MDA-MB-468
b
HGF (min):
c-Met
β1-integrin
LC3B
Merge / DAPI
MDA-MB-468
c
β1-integrin
c-Met
LC3B
Merge / DAPI
Y-Z
X-Z
MDA-MB-468
d
c-Met-β1 colocalisation with LC3B / total c-Met
HGF(min):
A549
e
c-Met
β1-integrin
LC3B
Merge
c-Met-β1-integrin:LC3B coloc: 13.2% ± 1.03
β1A
f
c-Met
β1-integrin
LC3B
Merge
c-Met-β1-integrin:LC3B coloc: 30.9% ± 2.56
M1268T
g
Active conformation β1-integrin / DAPI
c-Met / DAPI
LC3B / DAPI
Merge
cpdm
h
siRNA:
Cont
ATG5
LC3B
ATG5
Tubulin
A549
i
siRNA:
Cont
ATG13
LC3B
ATG13
Tubulin
A549
j
siRNA:
Cont
Beclin1
LC3B
Beclin1
Tubulin
A549
k
siRNA:
Cont
ATG5
HGF:
c-Met p145 p170
β1-integrin
ATG5
Tubulin
P-ERK 1/2
ERK 1/2
P-ERK1/2 / Tubulin:
1 2.0 ± 0.27 1 1.3 ± 0.17
*
NS
MDA-MB-468
l
P-c-Met / c-Met
NS
siRNA:
Cont
ATG5
A549
m
c-Met / Tubulin
NS
siRNA:
Cont
ATG5
A549
n
% HGF-555 uptake
NS
siRNA:
Cont
ATG5
A549
o
siRNA:
Cont
ATG5
min:
15
120
HGF:
ATG5
Tubulin
P-ERK1/2
P-ERK1/2 / Tubulin:
1
0.99 ± 0.05
1
0.47 ± 0.06
cpdm

Supplementary Figure 5 (related to Figure 5): c-Met and β1-integrin co-traffic on autophagy related endomembranes

a) Fold increases in colocalisation of c-Met-β1-integrin with EEA1, Rab4, Rab7 or LC3B, divided by total c-Met expression, upon 30 and 120 min HGF stimulation in MDA-MB-468 cells, normalised to the colocalisaiton values at 0 min (minimum of 5 pictures per condition, n=1).
b) Confocal section of adherent MDA-MB-468 cells stimulated with HGF for 0 and 120 min. Cells were stained for c-Met (blue), β1-integrin (red), LC3B (green) and DAPI (magenta). Bars: 10 µm.
c) Confocal sections of MDA-MB-468 cells stained for β1-integrin (green), c-Met (red), LC3B (blue) and DAPI (magenta) stimulated with HGF for 120 min. Orthogonal reconstructions of 10 serial confocal slices are shown (y-z and x-z axis with 1: β1-integrin, 2: c-Met, 3: LC3B, 4: merge of 1,2 and 3) alongside one z-slice taken in the middle of the cells. The perpendicular yellow lines on the section indicate from where the orthogonal views were built.
d) Fold increases in colocalisation of c-Met-β1-integrin with LC3B, divided by total c-Met expression, upon 15, 45 and 120 min HGF stimulation in A549 cells, normalised to the colocalisaiton values at 0 min (minimum of 8 pictures per condition, n=1).
ef) Confocal section of (e) β1A cells stimulated with HGF for 120 min or (f) M1268T c-Met expressing NIH3T3 cells stained for c-Met (red), β1-integrin (green) and LC3B (blue). Bar: 10 µm. Numbers are mean percentage colocalisation ± SEM (e) (n=1) (f) (n=3).
g) Confocal section of cpdm (Sharpin null) cells stimulated with HGF for 120 min, fixed and stained for c-Met (red), active-conformation β1-integrin (9EG7) (green), LC3B (blue) and DAPI (Magenta). Bar: 10 µm
hij) Western blots for LC3B, tubulin and (h) ATG5 (i) ATG13 or (j) Beclin1 in A549 cells transfected with control or (h) ATG5, (i) ATG13 or (j) Beclin1 siRNA and maintained in suspension.
k) Western blots for c-Met, β1-integrin, ATG5, tubulin, phospho-ERK1/2, and ERK1/2 in MDA-MB-468 cells, transfected with control or ATG5 siRNA and stimulated without (-) or with (+) HGF for 120 min in suspension. Numbers represent the mean phospho-ERK1/2 / tubulin ratios ± SEM with HGF normalised to the ratios without HGF (n=5).
lm) Quantifications of (5f) by densitometry. Graphs represent the mean ratios ± SEM of (m) phospho-c-Met / c-Met or (n) c-Met / tubulin upon HGF stmulation in cells transfected with ATG5 siRNA normalised to the ratios obtained in Control siRNA transfected cells (n=4).
n) Percentage of HGF-AlexaFluor-555 (HGF-555) uptake (mean red pixels per cell) ± SEM in A549 cells after 120 min in suspension, transfected with ATG5 siRNA normalised to the value obtained in cells transfected with control siRNA (n=3).
o) Western blots for phospho-ERK1/2, tubulin and ATG5 in cpdm (Sharpin null) MEFs, stimulated without (-) or with (+) HGF for 15 and 120 min in suspension after transfection with control or ATG5 siRNA. Numbers represent the mean phospho-ERK1/2 / tubulin ratios (± range of error) upon 15 and 120 min HGF, in cells transfected with ATG5 siRNA, normalised to the ratios obtained in cells transfected with control siRNA (n=2).
* $p<0.05$; NS: not significant.

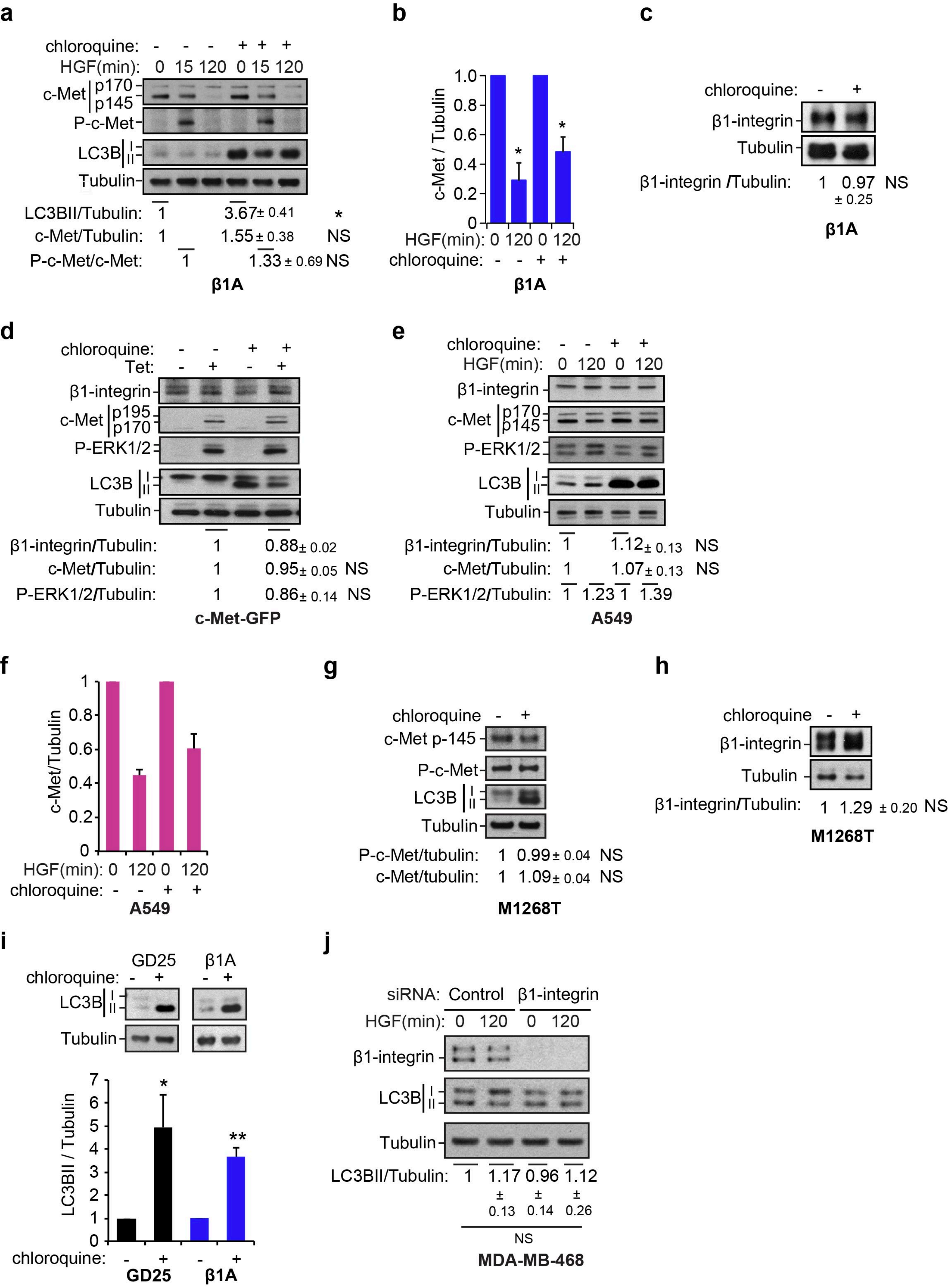
a
chloroquine: - - - + + +
HGF(min): 0 15 120 0 15 120
c-Met p170 p145
P-c-Met
LC3B I II
Tubulin
LC3BII/Tubulin: 1 3.67± 0.41 *
c-Met/Tubulin: 1 1.55 ± 0.38 NS
P-c-Met/c-Met: 1 1.33 ± 0.69 NS
β1A
b
c-Met / Tubulin
1.0 0.8 0.6 0.4 0.2 0
HGF(min): 0 120 0 120
chloroquine: - - + +
β1A
c
chloroquine: - +
β1-integrin
Tubulin
β1-integrin /Tubulin: 1 0.97 ± 0.25 NS
β1A
d
chloroquine: - - + +
Tet: - + - +
β1-integrin
c-Met p195 p170
P-ERK1/2
LC3B I II
Tubulin
β1-integrin/Tubulin: 1 0.88± 0.02
c-Met/Tubulin: 1 0.95± 0.05 NS
P-ERK1/2/Tubulin: 1 0.86± 0.14 NS
c-Met-GFP
e
chloroquine: - - + +
HGF(min): 0 120 0 120
β1-integrin
c-Met p170 p145
P-ERK1/2
LC3B I II
Tubulin
β1-integrin/Tubulin: 1 1.12± 0.13 NS
c-Met/Tubulin: 1 1.07± 0.13 NS
P-ERK1/2/Tubulin: 1 1.23 1 1.39
A549
f
c-Met/Tubulin
1 0.8 0.6 0.4 0.2 0
HGF(min): 0 120 0 120
chloroquine: - - + +
A549
g
chloroquine - +
c-Met p-145
P-c-Met
LC3B I II
Tubulin
P-c-Met/tubulin: 1 0.99± 0.04 NS
c-Met/tubulin: 1 1.09± 0.04 NS
M1268T
h
chloroquine - +
β1-integrin
Tubulin
β1-integrin/Tubulin: 1 1.29 ± 0.20 NS
M1268T
i
GD25 β1A
chloroquine: - + - +
LC3B I II
Tubulin
LC3BII / Tubulin
7 6 5 4 3 2 1 0
*
**
chloroquine: - + - +
GD25 β1A
j
siRNA: Control β1-integrin
HGF(min): 0 120 0 120
β1-integrin
LC3B I II
Tubulin
LC3BII/Tubulin: 1 1.17 0.96 1.12
± 0.13 ± 0.14 ± 0.26
NS
MDA-MB-468

Supplementary Figure 6 (related to Figure 5): Autophagy has no role on c-Met/β1-integrin signaling

a) Western blots for c-Met, phospho-c-Met, LC3B and tubulin in β1A cells treated with or without chloroquine (150 μM) for 4 h and stimulated with HGF (50ng/ml) for 0, 15 and 120 min. Numbers represent fold change + versus - chloroquine ± SEM (n=3) of LC3BII/tubulin and c-Met/tubulin levels at 0 min HGF and phospho-c-Met/c-Met levels at 15 min HGF, obtained by densitometric analysis.
b) Densitometric analyses of (a); c-Met/tubulin ratios at 120 min of HGF stimulation + or - chloroquine, normalised on ratios at 0 min, set at 1. Data are mean ± SEM (n=3).
c) Western blots for β1-integrin and tubulin in β1A cells treated wi-th or without chloroquine (150 μM) for 4 h. Numbers represent fold change + versus - chloroquine treatment ± SEM (n=3) of β 1-integrin/tubulin levels.
d) Western blots for β1-integrin, c-Met, phospho-ERK1/2, LC3B and tubulin in c-Met-GFP cells treated with or without chloroquine (150 μM) overnight and stimulated with Tet for 0 or 16 h. Numbers represent fold change ± SEM of β1-integrin/tubulin (n=2), c-Met/tubulin (n=3) and phospho-ERK1/2/tubulin (n=3) levels + Tet + chloroquine, normalised to + Tet - chloroquine, obtained by densitometric analysis.
e) Western blots for β1-integrin, c-Met, phospho-ERK1/2, LC3B and tubulin in A549 cells treated with or without chloroquine (150 μM) overnight and stimulated with HGF (100ng/ml) for 0 or 120 min. Numbers are fold change upon chloroquine versus no chloroquine ± SEM of β1-integrin/tubulin and c-Met/tubulin (n=3) at 0 min. Phospho-ERK1/2/tubulin ratios at 120 min HGF versus 0 min (set as 1) treated (+) or not (-) with chloroquine are also shown (n=1).
f) Densitometric analyses of (e); c-Met/tubulin ratios at 120 min HGF stimulation treated (+) or not (-) chloroquine, normalised on ratios at 0 min, set at 1. Data are mean ± range of error (n=2).
gh) Western blots for g) c-Met, phospho-c-Met, LC3B and tubulin or h) β1-integrin and tubulin in c-Met M1268T cells treated with (+) or without (-) chloroquine (150 μM) for 4 h. Numbers represent fold change + versus - chloroquine ± SEM of g) phospho-c-Met/tubulin (n=3), c-Met/tubulin (n=3), or h) β1-integrin/tubulin (n=3) levels, obtained by densitometric analysis.
i) Western blots of LC3B and tubulin in GD25 and β1A cells treated with (+) or without (-) chloroquine (150 μM) for 4 h. The Western blots are from the same membrane. Graph shows the LC3-BII/tubulin ratios in GD25 and β1A cells treated with or without chloroquine (150 μM) for 4 h obtained by densitometric analysis. Data are mean ± SEM (n=3), + versus - chloroquine.
j) Western blots for β1-integrin, LC3B and tubulin in MDA-MB-468 cells, transfected with control or β1-integrin siRNA for 72 h and stimulated with or without HGF (50ng/ml) for 120 min in suspension. Numbers represent fold change ± SEM of LC3BII/tubulin normalised to no HGF and no chloroquine treatment, obtained by densitometric analysis (n=3).
*p<0.05, **p<0.01, NS=not significant.

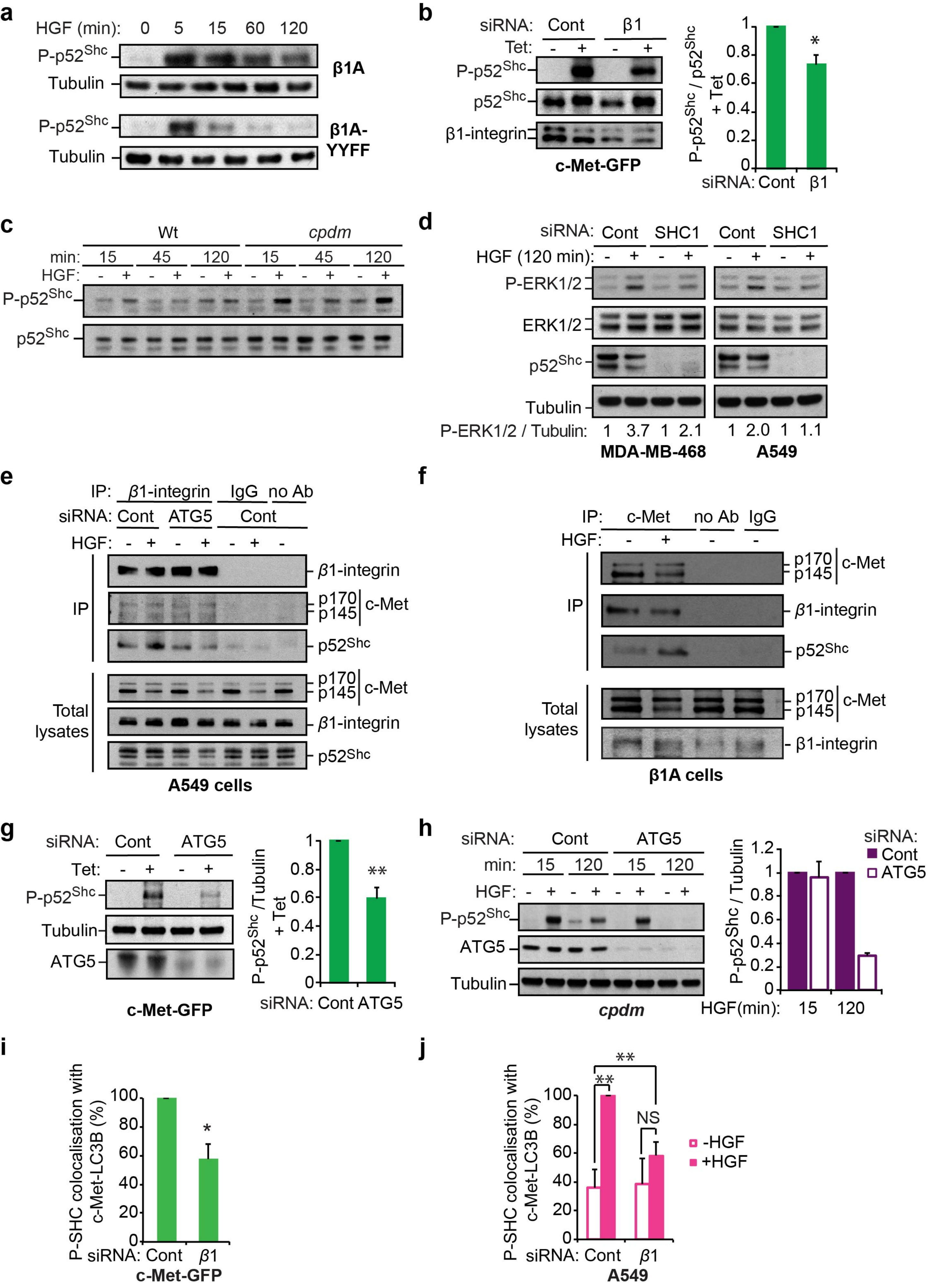
a
HGF (min): 0 5 15 60 120
P-p52Shc
Tubulin
β1A
P-p52Shc
Tubulin
β1A-YYFF
b
siRNA: Cont β1
Tet: - + - +
P-p52Shc
p52Shc
β1-integrin
c-Met-GFP
P-p52Shc / p52Shc + Tet
siRNA: Cont β1
c
Wt cpdm
min: 15 45 120 15 45 120
HGF:
P-p52Shc
p52Shc
d
siRNA: Cont SHC1 Cont SHC1
HGF (120 min):
P-ERK1/2
ERK1/2
p52Shc
Tubulin
P-ERK1/2 / Tubulin: 1 3.7 1 2.1 1 2.0 1 1.1
MDA-MB-468
A549
e
IP: β1-integrin IgG no Ab
siRNA: Cont ATG5 Cont
HGF:
IP
β1-integrin
p170
p145
c-Met
p52Shc
Total lysates
A549 cells
f
IP: c-Met no Ab IgG
HGF:
IP
c-Met
β1-integrin
p52Shc
Total lysates
β1A cells
g
siRNA: Cont ATG5
Tet:
P-p52Shc
Tubulin
ATG5
c-Met-GFP
P-p52Shc /Tubulin + Tet
siRNA: Cont ATG5
h
siRNA: Cont ATG5
min: 15 120 15 120
HGF:
P-p52Shc
ATG5
Tubulin
cpdm
P-p52Shc / Tubulin
siRNA:
Cont
ATG5
HGF(min): 15 120
i
P-SHC colocalisation with c-Met-LC3B (%)
siRNA: Cont β1
c-Met-GFP
j
P-SHC colocalisation with c-Met-LC3B (%)
NS
-HGF
+HGF
siRNA: Cont β1
A549

Supplementary Figure 7 (related to figure 6): β1-integrin plays the role of an adaptor to sustain c-Met signalling on ARE

a) Western blots for phospho-p52Shc and tubulin in β1A and β1A-YYFF cells stimulated with HGF for 0, 5, 15, 60 and 120 min.

b) Western blots for phospho-p52Shc, p52Shc and β1-integrin in c-Met-GFP cells, transfected with control (Cont) or β1-integrin (β1) siRNA and incubated without (-) or with (+) tetracycline (Tet) for 16 h. Graph represents phospho-p52Shc / p52Shc ratios ± SEM in presence of Tet in cells transfected with β1-integrin siRNA, normalised to the ratios obtained in cells transfected with control siRNA, obtained by densitometry (n=3).

c) Western blots for phospho-p52Shc and p52Shc in Wt and cpdm (Sharpin null) MEFs, stimulated without (-) or with (+) HGF for 15, 45 and 120 min in suspension.

d) Western blots for phospho-ERK1/2, ERK1/2, p52Shc and tubulin in MDA-MB-468 (left panel) and A549 (right panel), transfected with control or SHC1 siRNA and stimulated without (-) or with (+) HGF for 120 min in suspension. Numbers are the mean phospho-ERK1/2 / tubulin ratios upon HGF normalised to the ratios with no HGF, obtained by densitometric analysis (n=1).

e) Western blots for β1-integrin and p52Shc in A549 cells following immunoprecipitation with β 1-integrin, IgG control or no antibody (no Ab). Cells were stimulated without (-) or with (+) HGF for 120 min in suspension. Total β1-integrin and p52Shc levels in the cell lysates are shown.

f) Western blots for c-Met, β1-integrin and phospho-p52Shc following immunoprecipitation with c-Met B2 antibody, IgG control or no antibody (beads only) in β1A cells (same experiment as Fig. 1j). Cells were stimulated with HGF for 0 or 120 min. Total c-Met and β1-integrin levels in the cell lysates are shown.

g) Western blots for phospho-p52Shc, tubulin and ATG5 in c-Met GFP cells transfected with control or ATG5 siRNA and incubated without (-) or with (+) tetracycline (Tet) for 16 h. Graph represents the mean phospho-p52Shc / tubulin ratios ± SEM in cells transfected with ATG5 siRNA normalised to the ratios in cells transfected with control siRNA, in presence of Tet, obtained by densitometry (n=3).

h) Western blots for phospho-p52Shc, ATG5 and tubulin in cpdm (Sharpin null) MEFs, transfected with control or ATG5 siRNA and stimulated without (-) or with (+) HGF for 15 or 120 min in suspension. Graph represents the mean phospho-p52Shc / tubulin ratios ± range of error in presence of 15 or 120 min of HGF, in cells transfected with ATG5 siRNA normalised to the ratios in cells transfected with control siRNA, obtained by densitometry (n=2).

ij) Quantification of the percentage of phospho-SHC that colocalises with c-Met-LC3B in i) c-Met-GFP cells upon Tet and j) A549 cells with and without HGF for 120 min. Data are mean ± SEM (n=3).

* $p<0.05$; ** $p<0.01$.

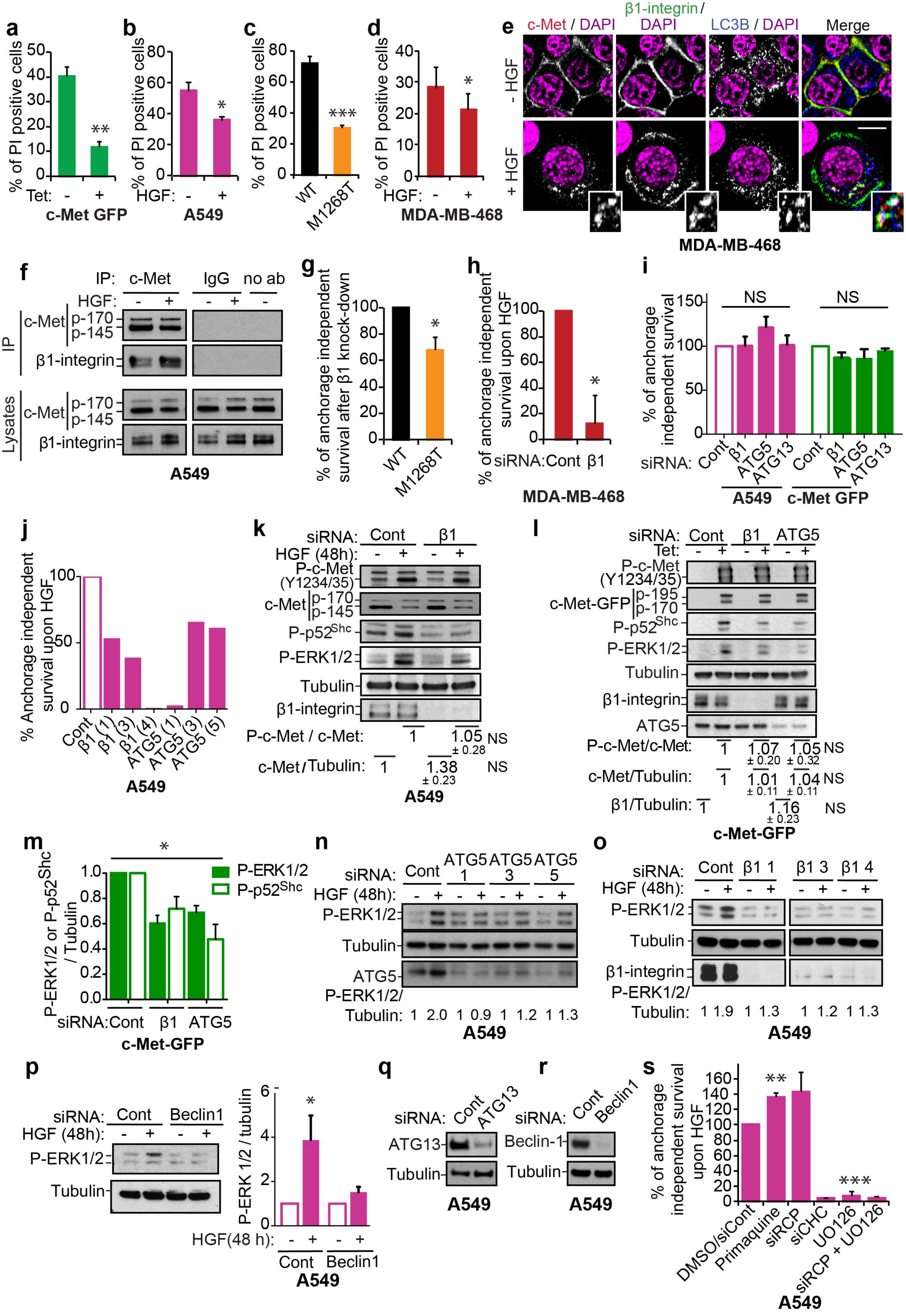
a
% of PI positive cells
Tet: - +
c-Met GFP
b
HGF: - +
A549
c
WT
M1268T
d
MDA-MB-468
e
c-Met / DAPI
β1-integrin / DAPI
LC3B / DAPI
Merge
- HGF
+ HGF
MDA-MB-468
f
IP: c-Met IgG no ab
HGF: - + - + -
IP
c-Met p-170 p-145
β1-integrin
Lysates
A549
g
% of anchorage independent survival after β1 knock-down
WT
M1268T
h
% of anchorage independent survival upon HGF
siRNA: Cont β1
MDA-MB-468
i
% of anchorage independent survival
NS
siRNA: Cont β1 ATG5 ATG13 Cont β1 ATG5 ATG13
A549
c-Met GFP
j
% Anchorage independent survival upon HGF
Cont
β1 (1)
β1 (3)
β1 (4)
ATG5 (1)
ATG5 (3)
ATG5 (5)
A549
k
siRNA: Cont β1
HGF (48h): - + - +
P-c-Met (Y1234/35)
c-Met p-170 p-145
P-p52Shc
P-ERK1/2
Tubulin
β1-integrin
P-c-Met / c-Met: 1 1.05 ± 0.28 NS
c-Met / Tubulin: 1 1.38 ± 0.23 NS
A549
l
siRNA: Cont β1 ATG5
Tet: - + - + - +
P-c-Met (Y1234/35)
c-Met-GFP p-195 p-170
P-p52Shc
P-ERK1/2
Tubulin
β1-integrin
ATG5
P-c-Met/c-Met: 1 1.07 ± 0.20 1.05 ± 0.32 NS
c-Met/Tubulin: 1 1.01 ± 0.11 1.04 ± 0.11 NS
β1/Tubulin: 1 1.16 ± 0.23 NS
c-Met-GFP
m
P-ERK1/2 or P-p52Shc / Tubulin
P-ERK1/2
P-p52Shc
siRNA: Cont β1 ATG5
c-Met-GFP
n
siRNA: Cont ATG5 1 ATG5 3 ATG5 5
HGF (48h): - + - + - + - +
P-ERK1/2
Tubulin
ATG5
P-ERK1/2/ Tubulin: 1 2.0 1 0.9 1 1.2 1 1.3
A549
o
siRNA: Cont β1 1 β1 3 β1 4
HGF (48h): - + - + - + - +
P-ERK1/2
Tubulin
β1-integrin
P-ERK1/2/ Tubulin: 1 1.9 1 1.3 1 1.2 1 1.3
A549
p
siRNA: Cont Beclin1
HGF (48h): - + - +
P-ERK1/2
Tubulin
P-ERK 1/2 / tubulin
HGF(48 h): - + - +
Cont Beclin1
A549
q
siRNA: Cont ATG13
ATG13
Tubulin
A549
r
siRNA: Cont Beclin1
Beclin-1
Tubulin
A549
s
% of anchorage independent survival upon HGF
DMSO/siCont
Primaquine
siRCP
siCHC
UO126
siRCP + UO126
A549

Supplementary Figure 8 (related to Figure 7): c-Met-β1-integrin cooperation on ARE mediates c-Met-dependent anchorage-independent cell survival

a-d) Mean percentage of propidium iodide (PI) positive cells ± SEM, assessed by flow cytometry. Cells were incubated in anchorage independent conditions; a) c-Met GFP cells without (-) or with (+) tetracycline (Tet) for 24 h (n=3); b) A549 without (-) or with (+) HGF for 48 h (n=3); c) Wt and M1268T c-Met expressing NIH3T3 cells for 24 h (n=3); d) MDA-MB-468 cells without (-) or with (+) HGF for 48 h (n=6).

e) Confocal sections of MDA-MB-468 incubated for 48 h in suspension without (-) or with (+) HGF. Cells were cytospun, fixed and stained for c-Met (red), β1-integrin (green), LC3B (blue) and DAPI (magenta). Bars: 10 μm.

f) Western blots for c-Met and β1-integrin following immunoprecipitations with c-Met, IgG control or no antibody (no ab) in A549 cells incubated without (-) or with (+) HGF for 48 h in suspension. Total β1-integrin and c-Met levels in the cell lysates are shown.

g) Mean percentage ± SEM of anchorage-independent survival after 24 h in suspension in Wt and M1268T c-Met expressing NIH3T3 cells upon transfection with β1-integrin, normalised to Wt c-Met expressing cells. Cells stained with propidium iodide were assessed by flow cytometry (n=3).

h) Mean percentage HGF-dependent anchorage-independent survival (or protection against anoikis) ± SEM in MDA-MB-468 cells transfected with β1-integrin (β1) siRNA, normalised to control (Cont) siRNA. Cells were stimulated with or without HGF for 48 h in suspension. Cells were stained with propidium iodide and the cell viability was analysed by flow cytometry (n=4).

i) Mean percentage ± SEM anchorage-independent survival of c-Met-GFP cells incubated in suspension for 24 h without tetracycline (Tet) (n=3) and A549 cells incubated in suspension for 48 h without HGF (n=6), following transfection with β1-integrin, ATG5 or ATG13 siRNA, normalised to the percentage obtained in cells transfected with control siRNA.

j) Percentage HGF- or tetracycline-dependent anchorage-independent survival (or protection against anoikis) in cells transfected with 3 individual β1-integrin (β1) or ATG5 siRNA oligos, normalised to control (Cont) siRNA. A549 cells were stimulated with HGF for 48 h in suspension. The HGF-dependent anchorage-independent survival was obtained by normalising the data with HGF to no HGF. Cells were stained with propidium iodide and the cell viability was analysed by flow cytometry (n=1).

k) Western blots for phospho-c-Met and c-Met, phospho-ERK1/2, phospho-p52Shc, tubulin and β 1-integrin in A549 cells, transfected with control (Cont) or β1-integrin (β1) siRNA and stimulated without (-) or with (+) HGF for 48 h in suspension. Numbers underneath represent the mean ratios ± SEM of phospho-c-Met / c-Met in presence of HGF and of c-Met / tubulin in absence of HGF in cells transfected with β1-integrin siRNA, normalised to the ratios in cells transfected with control siRNA. Data were obtained by densitometry (n=3).

l) Western blots for phospho-c-Met, c-Met, phospho- p52Shc, tubulin, β1-integrin and ATG5 in c-Met GFP cells, transfected with control, β1-integrin or ATG5 siRNA and stimulated without (-) or with (+) tetracycline (Tet) for 24 h in suspension. The numbers underneath represent the mean ratios ± SEM of phospho-c-Met / c-Met or c-Met / tubulin in presence of Tet (top lines) and of β1-integrin / tubulin in absence of Tet (bottom line) in cells transfected with β1-integrin or ATG5 siRNA normalised to the ratios in cells transfected with siRNA control. Data were obtained by densitometry (n=3)

m) Quantifications of l) Graph represents the mean phospho-ERK1/2 / tubulin and phospho-p52Shc / tubulin ratios ± SEM in presence of tetracycline (Tet), obtained by densitometric analysis, in cells transfected with β1-integrin or ATG5 siRNA, normalised to the ratios ontained in control siRNA transfected cells (n=3).

no) Western blots for phospho-ERK1/2, tubulin and (n) ATG5 or (o) β1-integrin in A549 cells, transfected with control (Cont) siRNA or 3 individual (n) ATG5 (1, 3 and 5) or (o) β1-integrin (1, 3 and 4) siRNA oligos and stimulated without (-) or with (+) HGF for 48 h in suspension. Numbers represent mean phospho-ERK1/2 / tubulin ratios at 120 min with HGF normalised to no HGF (n=1). (o) The Western blots are from the same membrane and the same exposure for each antibody.

p) Western blots for phospho-ERK1/2 and tubulin in A549 cells transfected with control or Beclin1 siRNA (oligo 2) and stimulated without (-) or with (+) HGF for 48 h in suspension. Graph represents the mean fold increases of phospho-ERK1/2 / tubulin upon HGF versus no HGF ± SEM (n=3), obtained by densitometric analysis, in cells transfected with control or Beclin1 siRNA.

s) Mean percentage HGF-dependent anchorage-independent survival (or protection against anoikis) ± SEM or ± range of error in A549 cells, incubated for 48 h with HGF in suspension, following treatment with Primaquine (60 µM)(n=3), or transfection with Rab-coupling protein (RCP)(n=3) or Clathrin heavy chain (CHC)(n=1) siRNA or treatment with U0126 (10 µM)(n=3) or tranfection with RCP siRNA and U0126 treatment combined (n=1). Ratios are normalised to the ratios in cells treated wtih DMSO or transfected with control siRNA. Cells were stained with propidium iodide solution and assessed by flow cytometry.
* $p<0.05$; **$p<0.01$; ***$p<0.001$; NS: not significant.

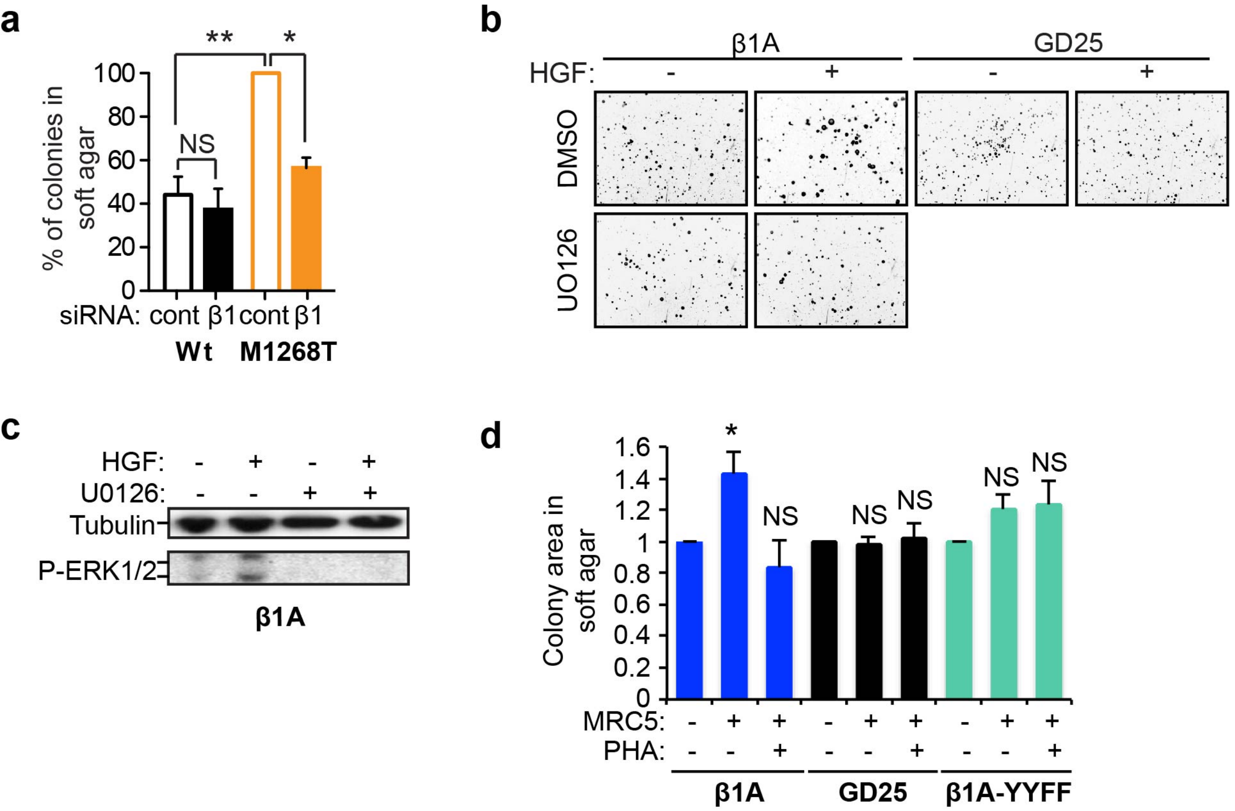

Supplementary Figure 9 (related to Figure 8): c-Met-β1-integrin intracellular cooperation mediates c-Met-dependent anchorage-independent growth, in vivo tumourigenesis and invasion

a) Percentage of the mean number of colonies ± SEM at day 6 in soft agar of Wt and M1268T c-Met expressing NIH3T3 cells transfected with control (cont) or β1-integrin (β1) siRNA. Data are normalised to the value in M1268T c-Met expressing cells transfected with control siRNA (n=3, each experiment done in triplicate).

b) Pictures of β1A and GD25 colonies at day 13 of growth in soft agar. From day 8, HGF (14 ng/ml) was added to the medium, or not, every other day as well as DMSO or UO126 (10 μM).

c) Western blots of phospho-ERK1/2 and tubulin from the colonies of β1A cells grown in soft agar in (b).

d) Mean total colony area of β1A, GD25 and β1A-YYFF cells at day 13 of growth in soft agar. From day 8, the cultures were incubated with or without MRC5 conditioned media and treated with DMSO or PHA-665752 (PHA, 100 nM). Data are normalised to the values obtained in cells incubated without MRC5 conditioned media and treated with DMSO (n=3, done in triplicate).

* $p<0.05$; ** $p<0.01$; NS: not significant.

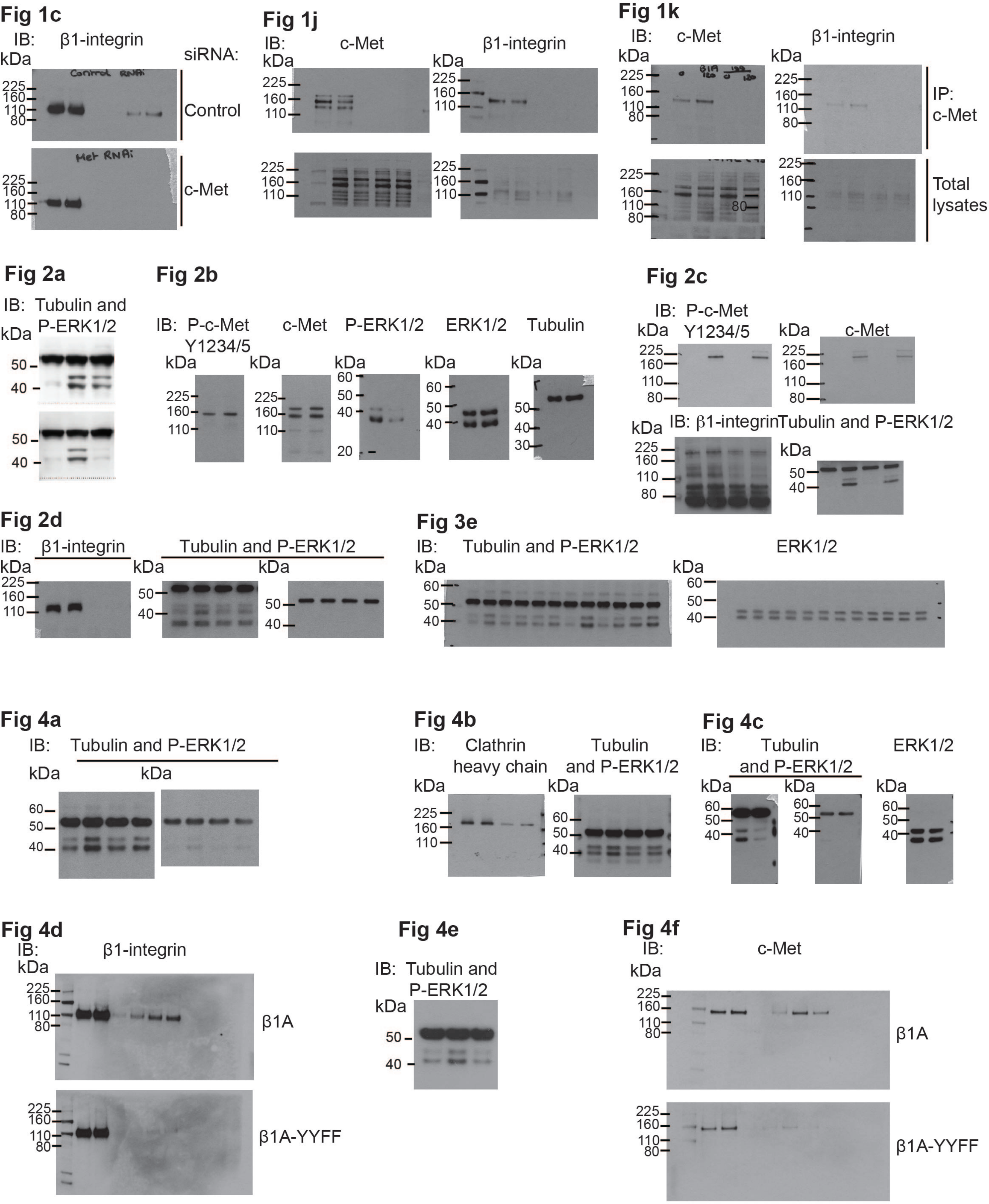

Supplementary Figure 10- Uncropped blots of the indicated figures

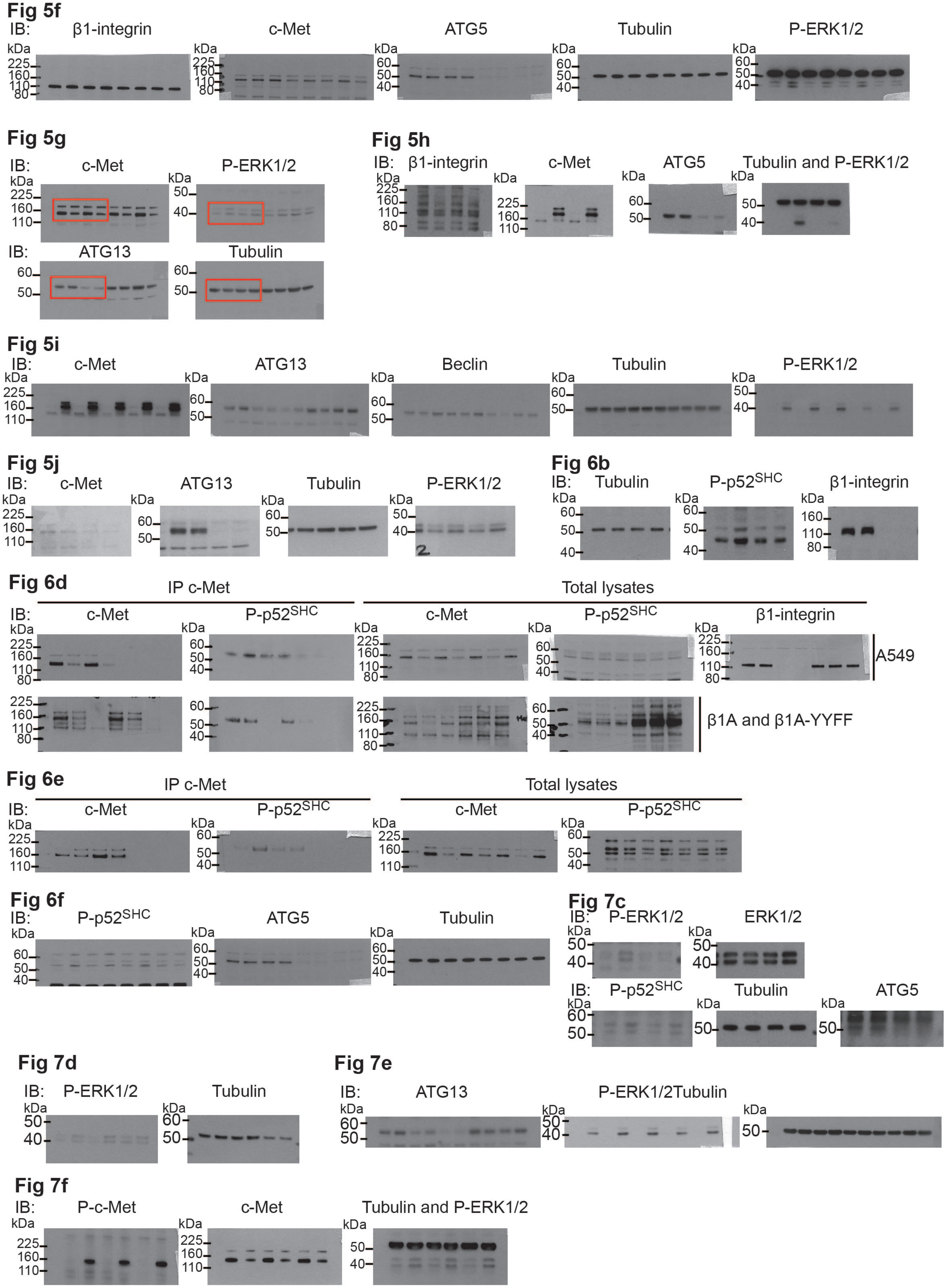

Supplementary Figure 11- Uncropped blots of the indicated figures

Supplementary Table 1: siRNA sequences

siRNAI	Target Sequence	Purchased from
**Mouse β1-integrin (used as a pool or individual oligos)**	GCACAGAUCCCAAGUUUCA (oligo 3)	Dharmacon
	GAACGGAUUUGAUGAAUGA (oligo 1)	
	CAAGAGGGCUGAAGAUUAC (oligo 4)	
**Human β1-integrin (used as a pool or individual oligos)**	GAACAGAUCUGAUGAAUGA (oligo 1)	Dharmacon
	GAAGGGAGUUUGCUAAAUU (oligo 3)	
	CCACAGACAUUUACAUUAA (oligo 4)	
**Mouse c-Met (used as a pool)**	GGACUUUGCUGGACAAUGA	Dharmacon
	GAACAGCGAGCUAAAUAUA	
	GGGAAGAAGUGUUUAAUAU	
	CCAGAGACAUGUACGAUAA	
**Human ATG5 (used as a pool or individual oligos)**	GGAAUAUCCUGCAGAAGAA (oligo 1)	Dharmacon
	CAUCUGAGCUACCCGGAUA (oligo 3)	
	GACAAGAAGACAUUAGUGA (oligo 5)	
	CAAUUGGUUUGCUAUUUGA (olgo 6)	
**Human Beclin1 (used as a pool or individual oligos)**	GGAUGACAGUGAACAGUUA (oligo 2)	Dharmacon
	UAAGAUGGGUCUGAAAUUU (oligo 3)	
	GCCAACAGCUUCACUCUGA (oligo 4)	
	UUGAAAACCAGAUGCGUUA (oligo 17)	
**Human ATG13 (used as a pool or individual oligos)**	GAAAGGACCUGGACAAGUU (oligo 1)	Dharmacon
	CCAUGGAGCUGGAAAUAUG (oligo 2)	
	GAAGAAUGUCCGCGAGUUU (oligo 3)	
	GUUUGGAAAUACCGAGCUA (oligo 17)	
**Human RCP**	CAAACAGAAGGAAACGAUA	Dharmacon
**Human SHC1**	GAGUUGCGCUUCAAACAAU (oligo 4)	Dharmacon
	CAGCCGAGUAUGUCGCCUA (oligo 19)	
**Human β1-integrin**	AAGAAGGCTCGAGAGTCCTAT	Qiagen
**The AllStars Neg. Control siRNA**	No sequence provided	Qiagen
